# Supplementary material for: Identification of photoperiod-induced specific miRNAs in the adrenal glands of Sunite sheep (Ovis aries)
Source: Front Vet Sci. 2022 Jul 22;9:888207. doi: 10.3389/fvets.2022.888207 (PMC9354845; doi:10.3389/fvets.2022.888207)
Supplement: Supplementary file 1 [file Data_Sheet_1.ZIP › Supplementary file/miRDeep2 file.zip!/miRDeep2 file/Know_Structure/SPLP42A3/oar-mir-29a.pdf]

miRBase precursor : oar-mir-29a
Total read count : 80156
oar-miR-29a read count : 80122
remaining reads : 34

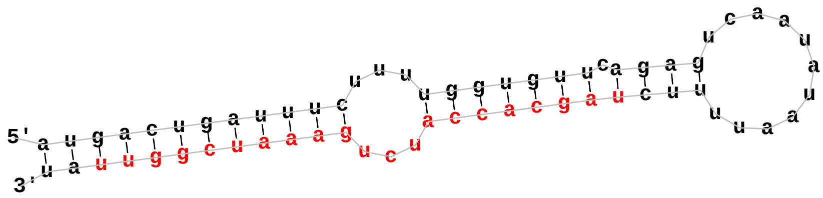

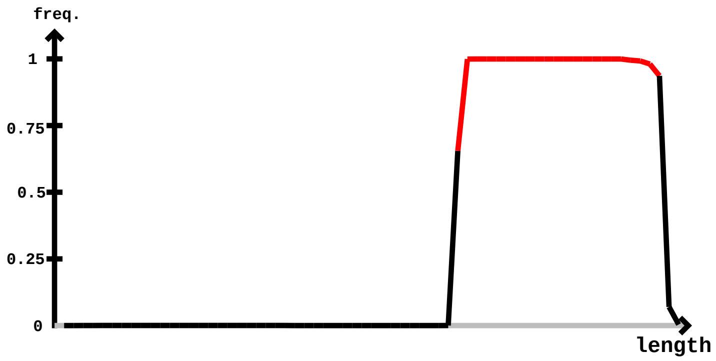

oar-miR-29a

```
5'- augacugauuucuuuugguguucagagucaauauaauuuucuagcaccaucugaaaucgguuau   -3'    exp
    ((((((((((((...(((((((.((((...........)))))))))))...))))))))))))    reads      mm         sample
    .ugacugauuucuuuugguguu..........................................    5          0          seq
    .ugacugauuucuuuugguguuc.........................................    15         0          seq
    ...acugauuucuuuugguguuc.........................................    12         0          seq
    ......................................uucuagcaccaucugaaaucggu...    2          0          seq
    .......................................ucuagcaccaucugaaauc......    1          0          seq
    .......................................ucuagcaccaucugaaaucg.....    1          0          seq
    .......................................ucuagcaccaucugaaaucgg....    4          0          seq
    .......................................ucuagcaccaucugaaaucggu...    1          0          seq
    .......................................ucuagcaccaucugaaaucgguu..    7          0          seq
    .......................................ucuagcaccauUugaaaucgguu..    1          1          seq
    ........................................cuaCcaccaucugaaau.......    1          1          seq
    ........................................cuagcaccaucugaaau.......    7          0          seq
    ........................................cuagcaccaucugaaGuc......    1          1          seq
    ........................................cuagcaccaucugaaauA......    5          1          seq
    ........................................cuagcaccaucugaaauc......    86         0          seq
    ........................................cuagcaccaCcugaaauc......    1          1          seq
    ........................................cuagcaccaucugaaaucg.....    58         0          seq
    ........................................cuagcaccaucugaaaucgg....    192        0          seq
    ........................................cuagcaccauUugaaaucggu...    15         1          seq
    ........................................cuagcaccaucugaaaucggu...    1284       0          seq
    ........................................cuagcaccaucugaaaucgguu..    50283      0          seq
    ........................................cuagcaccauUugaaaucgguu..    176        1          seq
    ........................................cuagcaccaucugaaaucgguua.    329        0          seq
    ........................................cuagcaccaucugaaaucgguuau    57         0          seq
    .........................................uagcaccaucugaaauU......    20         1          seq
    .........................................uaUcaccaucugaaauc......    2          1          seq
    .........................................uagcaccaucugaaauA......    5          1          seq
    .........................................uagcaccaucugaaauG......    1          1          seq
    .........................................uGgcaccaucugaaauc......    1          1          seq
    .........................................uagcaccaCcugaaauc......    1          1          seq
    .........................................uagcaccaucugaaauc......    238        0          seq
    .........................................uagcaccauUugaaaucg.....    10         1          seq
    .........................................uagcaccaucugaaaucg.....    158        0          seq
    .........................................uagcaccauUugaaaucgg....    32         1          seq
    .........................................uagcaccaucugaaaucgg....    692        0          seq
```

oar-miR-29a

```
augacugauuucuuuugguguucagagucaauauaauuuucuagcaccaucugaaaucgguuau
.........................................uagcaccauUugaaaucggu...    242     1    seq
.........................................uagcaccaucugaaaucggu...    2009    0    seq
.........................................uagcaccaucugaaaucgguu..    18335   0    seq
.........................................uagcaccauUugaaaucgguu..    615     1    seq
.........................................uagcaccauUugaaaucgguua.    236     1    seq
.........................................uagcaccaucugaaaucgguua.    4726    0    seq
.........................................uagcaccauUugaaaucgguuau    9       1    seq
.........................................uagcaccaucugaaaucgguuau    252     0    seq
..........................................agcaccaucugaaaucgg....    1       0    seq
..........................................agcaccaucugaaaucggu...    2       0    seq
..........................................agcaccaucugaaaucgguu..    18      0    seq
..........................................agcaccaucugaaaucgguua.    3       0    seq
..........................................agcaccaucugaaaucgguuau    1       0    seq
...........................................gcaccaucugaaaucgguu..    2       0    seq
............................................caccauUugaaaucgguuau    1       1    seq
```
